# Factors Influencing the Quality of Women’s Sexual Life: A Study of Polish Female Students

**DOI:** 10.3390/healthcare13243278

**Published:** 2025-12-13

**Authors:** Maciej Stokłosa, Iga Florczyk, Gniewko Więckiewicz, Karolina Kiersten, Magdalena Piegza, Robert Pudlo

**Affiliations:** 1 Department and Clinic of Psychiatry, Faculty of Medical Sciences in Zabrze, Medical University of Silesia, Pyskowicka 47-51, 42-612 Tarnowskie Góry, Poland; iga.florczyk@gmail.com (I.F.); gniewko.wieckiewicz@gmail.com (G.W.); mpiegza@sum.edu.pl (M.P.); rpudlo@sum.edu.pl (R.P.); 2 Department of Gynaecology, Obstetrics and Oncological Gynaecology Faculty of Medical Sciences in Zabrze, Medical University of Silesia, Stefana Batorego 15, 41-902 Bytom, Poland; karolina.kiersten@gmail.com

**Keywords:** female sexual function, FSFI, students

## Abstract

**Background/Objectives:** Women’s sexual quality of life is a multidimensional construct shaped by individual, psychological, relational, and health-related factors. This exploratory cross-sectional study aimed to identify selected determinants of sexual functioning in young women, with a particular focus on partner relationships and sexual dysfunction symptoms within the couple. **Methods:** Data from 199 female university students aged 18–30 years, recruited via Facebook, were analyzed. Participants completed the Female Sexual Function Index (FSFI) and an author-designed questionnaire assessing sociodemographic variables, relationship characteristics, and self-perceived sexual difficulties in themselves and their partners. Descriptive statistics, bivariate analyses, and multivariable linear regression models were used to examine factors associated with the FSFI total and domain scores. **Results:** In this self-selected, non-representative sample, over 75% of women reported at least one self-perceived sexual difficulty, while 35.2% obtained FSFI scores at or below the established cutoff, indicating an increased risk of female sexual dysfunction rather than a confirmed diagnosis. In multivariable analysis, higher intercourse frequency, greater overall sexual satisfaction in the last 12 months, and fewer self-reported dysfunction symptoms emerged as the strongest independent predictors of higher FSFI total scores. Women who perceived premature ejaculation in their male partners tended to report lower orgasm and satisfaction domain scores, although this perception was not independently associated with the FSFI total score after adjustment for individual and relationship factors. **Conclusions:** These findings underline the role of both individual and relational factors in young women’s sexual functioning and support a holistic, couple-centred perspective in sexual health assessment.

## 1. Introduction

The sexual life of young women represents an important area of research in the context of reproductive health, psychological well-being, and interpersonal relationships. Previous studies in the Polish population have highlighted the significant role of educational, environmental, and psychological factors in shaping women’s sexual functioning. Within the student population—composed predominantly of young adults—everyday life is increasingly shaped by dynamic social, educational, and technological changes, including the widespread use of digital technologies in learning, communication, and interpersonal relationships [1].

According to the World Health Organization (WHO), sexuality is an integral component of individual health, encompassing physical, emotional, mental, and social well-being, and not merely the absence of disease or dysfunction [2]. A meta-analysis of recent studies confirmed the central role of the Female Sexual Function Index (FSFI) in the epidemiology of sexual disorders, with prevalence rates ranging from 5.5% to as high as 77% [3]. Studies have also demonstrated a strong association between FSFI outcomes and psychological well-being. Higher levels of sexual satisfaction among women correlate with fewer depressive symptoms and more satisfying partner relationships, suggesting that sexual well-being may play a preventive role in mental health. At the same time, depressive symptoms themselves have been shown to negatively affect sexual desire, arousal, and satisfaction, indicating a bidirectional relationship between sexual health and psychological well-being [4,5].

Over the past few decades, approaches to women’s sexuality—both clinical and socio-cultural—have undergone significant evolution. The groundbreaking work of Masters and Johnson in the 1960s laid the foundations for the diagnosis and treatment of female sexual dysfunctions, while successive editions of the Diagnostic and Statistical Manual of Mental Disorders (DSM, 1952–2013) gradually redefined conditions such as dyspareunia, anorgasmia, and desire disorders, reflecting increasingly precise and inclusive conceptualizations of sexual functioning and its disorders [6,7].

Research conducted in Poland has shown that university students’ sexual lives are shaped by temperament and relationship satisfaction [8]. Other influential factors include socioeconomic status and stage of education [9]. Broader determinants of sexual life and satisfaction also include the COVID-19 pandemic, during which heightened anxiety and social isolation significantly affected sexual well-being [10]. Lifestyle factors such as physical activity, alcohol consumption, and tobacco use have been linked to psychosexual health and the occurrence of dysfunctions [9,11]. National analyses further indicate that approximately 19% of women aged 18–24 experience sexual dysfunctions, highlighting their relevance in this age group [12].

Limited access to comprehensive sexual education within the Polish school system continues to shape young women’s knowledge, influencing awareness of their sexuality, patterns of sexual behavior, and overall quality of sexual life [13]. The absence of systematic sexual education and the persistence of cultural taboos foster risky sexual behaviors and reduce awareness of responsible contraception, as well as early recognition of sexual difficulties [14].

Although female sexual functioning has been investigated in various Polish and Central/Eastern European populations, most FSFI-based studies have focused on clinical groups or broadly defined samples of women. In contrast, fewer recent studies have specifically targeted female university students and simultaneously considered women’s own sexual difficulties, their perceptions of male partner sexual dysfunctions, and brief global indicators of sexual satisfaction within a single analytical framework [15,16,17]. The present exploratory study addresses these gaps by examining individual, relational, and partner-related correlates of sexual functioning among a self-selected, non-representative sample of Polish female university students. We focus on the prevalence of dysfunction-level FSFI scores and self-reported sexual difficulties in this group, the associations between relationship status, sexual activity patterns, and perceived male partner sexual dysfunctions (including premature ejaculation), as well as women’s FSFI outcomes, and the relationship between a single-item measure of overall sexual satisfaction and FSFI scores.

The aim of this article is therefore not to provide population-level estimates, but to generate hypotheses and highlight clinically relevant patterns in a specific student population. Specifically, this study addressed the following research questions: (RQ1) What is the prevalence of dysfunction-level FSFI scores and self-reported sexual difficulties in this sample of Polish female university students? (RQ2) How are relationship status, sexual activity patterns, and perceived partner sexual dysfunctions—including premature ejaculation—associated with women’s sexual functioning as measured by the FSFI? (RQ3) Which factors independently predict FSFI total scores in multivariable analysis? Based on prior literature, we hypothesized the following: (H1) Dysfunction-level FSFI scores and self-reported sexual difficulties would be relatively common in this population. (H2) Women in stable relationships and those who do not perceive sexual dysfunctions in their partners would report higher sexual satisfaction and FSFI scores. (H3) Both women’s own sexual difficulties and perceived premature ejaculation in their male partners would be independently associated with lower FSFI total scores after controlling for sociodemographic and relational variables.

## 2. Materials and Methods

### 2.1. Study Design and Participants

This study is part of a project on the sexuality of Polish students called “PolSex2024”, the goal of which was to describe the sexual life of Polish students. In this article, we specifically aim to strongly emphasize the symptoms of sexual dysfunctions and the quality of sexual life. The study employed a cross-sectional, exploratory design utilizing an online survey method (Google Forms, Google LLC, Mountain View, CA, USA.) based on a non-probability convenience sampling technique, specifically chosen to ensure the highest possible level of respondent anonymity. Data collection took place from March to November 2024.

The recruitment process involved a multi-stage procedure. First, the 20 largest universities in Poland were randomly selected. Subsequently, administrators of social media groups (Facebook, Meta Platforms, Inc., Menlo Park, CA, USA) associating students from specific years and fields of study at these universities were contacted. Distribution of the survey link occurred only after obtaining the administrator’s explicit consent.

The inclusion criteria for the study were as follows: (1) status of a university student in Poland, (2) female gender assigned at birth, (3) age ≥ 18 years, (4) having undergone sexual initiation, and (5) providing informed consent.

Exclusion criteria included (1) incomplete questionnaire responses, (2) lack of sexual initiation, and (3) male gender.

### 2.2. Measures

The research tool consisted of three distinct sections:

1. Custom Sociodemographic Questionnaire: This section collected baseline characteristics of the participants, including age, place of residence, education level, religious practices, and current relationship status.
2. Sexual Life Questionnaire: An original set of questions designed to evaluate the following:
  ○ Sexual behaviors: frequency of sexual intercourse and number of sexual partners.
  ○ Self-reported symptoms: presence of sexual dysfunction symptoms experienced by the respondent (e.g., lack of desire, orgasmic difficulties).
  ○ Partner’s symptoms: presence of sexual dysfunction symptoms in the partner as observed and reported by the respondent (e.g., premature ejaculation, erectile dysfunction).
3. Female Sexual Function Index (FSFI): A 19-item standardized self-report instrument used for the multidimensional assessment of female sexual function over the past four weeks [18]. The scale evaluates six key domains: Desire, Arousal, Lubrication, Orgasm, Satisfaction, and Pain. Individual domain scores are calculated by multiplying the sum of sub-items by a domain factor, and the total score ranges from 2 to 36. Higher scores indicate better sexual functioning. A total score of ≤26.55 was adopted as the clinical cutoff for identifying women at risk of clinically significant sexual dysfunction [19].

The original components of the survey were validated by two independent experts in the field of sexology.

### 2.3. Ethical Considerations

Participation in the study was voluntary. All respondents were informed about the study’s purpose and provided digital informed consent before accessing the questionnaire. According to Decision No. BNW/NWN/0052/KB/213/23 of the Bioethics Committee of the Medical University of Silesia, formal ethical approval was waived for this non-interventional online survey study.

### 2.4. Statistical Analysis

Prior to analysis, the dataset was screened for missing values. Incomplete questionnaires were excluded from the study (listwise deletion). A post hoc power analysis was conducted using G*Power software (version 3.1.9.7, Heinrich-Heine-Universität Düsseldorf, Düsseldorf, Germany). With a final sample size of 199 and a significance level of α = 0.05, the study achieved > 80% statistical power to detect small-to-medium effect sizes (correlation coefficients r > 0.20 and regression effect sizes f^2^ > 0.06), confirming that the sample size was sufficient for the planned analyses.

Statistical analyses were performed using Statistica software (version 13.3, StatSoft, Kraków, Poland). Continuous variables are presented as means ± standard deviations (SDs) or medians, while categorical variables are expressed as absolute numbers and percentages. The Shapiro–Wilk test was used to assess the normality of the distribution. Due to the non-normal distribution of the majority of the data, non-parametric tests were employed.

Differences between two independent groups were analyzed using the Mann–Whitney U test, and comparisons involving more than two groups were conducted using the Kruskal–Wallis ANOVA, followed by appropriate post hoc tests. Correlations between continuous variables were evaluated using Spearman’s rank correlation coefficient. Comparisons of qualitative variables were performed using the Chi-square test.

To address the issue of multiple comparisons and control the risk of Type I error, the Benjamini–Hochberg procedure was applied to control the false discovery rate (FDR). Significance thresholds were adjusted accordingly.

To identify independent factors affecting the FSFI score, a multivariate linear regression analysis was performed. Variables that showed potential associations in the univariate analysis (*p* < 0.15) were considered for the model. Prior to the final model construction, collinearity diagnostics were conducted using tolerance and variance inflation factor (VIF). To ensure model stability and avoid redundancy, variables exhibiting high multicollinearity were excluded from the final multivariate model (all retained VIFs < 1.4).

For all statistical tests, a *p*-value of < 0.05 was considered statistically significant.

## 3. Results

A total of 368 survey responses were collected from students, from which 4 incorrectly completed questionnaires were removed. Subsequently, 315 respondents who had undergone sexual initiation were identified. Of these, 63.2% were individuals assigned female at birth, who were the subject of the present analysis.

### 3.1. Sociodemographic Factors

The characteristics of the study population are presented in Table 1. In total, 199 women were included in the analysis. The mean age of the respondents was 26.2 ± 6.2 years. A similar percentage of respondents was recorded in terms of the size of the city of origin, while in the case of current residence, a large part of the population—over 40%—lives in cities with over 200,000 inhabitants, which corresponds to academic centers. As many as 30% of respondents lived in their family home. 12% of them had at least one child. 22% of participants declared that they follow the principles of their religion in life, all of whom were Roman Catholic.

In summary, the study group was characterized by a predominance of women residing in large academic centers and declaring religious affiliation, which reflects the specific profile of the recruited sample rather than the general student population.

### 3.2. Sexual Orientations and Gender Identification

Nearly 17% of the women identified as bisexual, and 2% declared a homosexual orientation. 4.5% of the respondents reported identifying with a gender other than the one assigned at birth. Specifically, among the participants assigned female at birth, 6 identified as men and 3 as non-binary (3% and 1.5%, respectively). The characteristics of the population in terms of gender identification, sexual orientation, and being in a relationship are described in Table 2.

### 3.3. Relationship Status and Sexual Functioning

Notably, 82% of female students had one regular sexual partner, while 1.5% reported having multiple regular sexual partners. At the same time, only slightly more than half of the respondents were in a stable relationship, and 10% were married. Among those not in a stable relationship, 61% had one regular sexual partner, and 3% had multiple regular sexual partners. Over 60% of the respondents were engaged in sexual intercourse at least once a week, and 17% less than once a month. A higher frequency of intercourse was observed among those in a stable relationship (mean: 3.0 ± 1.0 vs. 2.2 ± 1.4; Mann–Whitney U Test; z = 4.8; *p* < 0.0001). Respondents in a stable relationship were significantly more satisfied with their sex life, both in the context of the last 12 months (mean: 4.1 ± 1.0 vs. 3.4 ± 1.2; Mann–Whitney U Test; z = 4.1; *p* < 0.0001) and over their entire lifetime (mean: 4.0 ± 0.9 vs. 3.6 ± 1.1; z = 3.1; *p* = 0.002), which is also reflected in the FSFI sexual satisfaction domain (mean: 5.0 ± 1.1 vs. 4.4 ± 1.3; z = 3.7; *p* = 0.0002). Similarly, being in a stable relationship was associated with higher scores on the FSFI scale in the domains of arousal (mean: 4.9 ± 1.0 vs. 4.3 ± 1.7; z = 2.3; *p* = 0.02), orgasm (mean: 4.4 ± 1.3 vs. 3.7 ± 1.8; z = 2.6; *p* = 0.009), and the total FSFI score (mean: 28.5 ± 5.0 vs. 25.3 ± 7.2; z = 3.4; *p* = 0.0007). A detailed analysis between relationship types (Kruskal–Wallis test; H = 31.2; *p* < 0.001) revealed a significantly higher age among married women compared to those in a stable, non-marital relationship (mean: 32.8 ± 6.5 vs. 24.6 ± 4.7; post hoc; z = 5.6; *p* < 0.0001) and those not in a stable relationship (mean: 32.8 ± 6.5 vs. 26.2 ± 6.5; post hoc; z = 4.5; *p* < 0.0001). This indicates that the only observed difference between relationship types was age, as a detailed analysis showed no significant variance between being married versus being in a partnership regarding the frequency of intercourse, sexual life satisfaction, or any FSFI domains. No statistically significant difference was observed in the prevalence of sexual dysfunction symptoms between respondents who were in a stable relationship and those who were not.

### 3.4. Sexual Satisfaction

A strong positive correlation was observed between sexual life satisfaction and the frequency of intercourse, with the correlation being stronger for satisfaction over the last 12 months than for lifetime satisfaction (Spearman’s Rank Correlation; r = 0.47 and r = 0.27, respectively; *p* < 0.0001). Furthermore, sexual life satisfaction significantly correlated with the corresponding FSFI domains and the total FSFI score, with stronger associations also observed for the assessment of the last 12 months compared to the entire lifetime. The strongest correlation was found between satisfaction over the last 12 months and the FSFI ‘satisfaction’ domain (r = 0.73; *p* < 0.0001), which indicates that even a simple 5-point Likert scale for satisfaction shows strong associations with FSFI scores and may be useful as a preliminary screening indicator of subjective sexual satisfaction, although it requires further validation.

### 3.5. Symptoms of Sexual Dysfunction

Subsequent analyses addressed the second research question regarding the associations between relationship status, sexual activity patterns, perceived partner dysfunctions, and women’s sexual functioning. Over 75% of the respondents experienced symptoms of at least one sexual dysfunction. More than half of the respondents had difficulty achieving orgasm during intercourse, nearly half reported dyspareunia, and just over 40% manifested decreased sexual interest.

### 3.6. Frequency of Intercourse and Symptoms of Sexual Dysfunction

A correlation was observed between a higher frequency of intercourse and a lower number of sexual dysfunction symptoms in respondents (Spearman’s rank correlation; r = −0.15; *p* = 0.034). The frequency of intercourse was analyzed in relation to the presence of specific sexual dysfunction symptoms, revealing less frequent intercourse in women who reported decreased sexual interest (Mann–Whitney U Test; Z = 2.98; *p* = 0.0028) and in students who reported problems with achieving orgasm (Z = 2.17; *p* = 0.029).

### 3.7. FSFI

The mean score on the FSFI was 27.1 ± 6.3, with a median of 28.3 (maximum possible score of 36). A score of 26.55 or less on the FSFI scale was used as a cutoff indicating an increased risk of clinically significant sexual dysfunction, which was observed in 35.2% of respondents. Table 3 presents the characteristics of sexual dysfunction symptoms in both the respondents and their partners, categorized into two groups based on the cutoff point: with and without clinically significant sexual dysfunction according to the FSFI. Respondents classified into the group with clinically significant sexual dysfunction according to the FSFI had a significantly lower number of partners in the last 12 months (Mann–Whitney U Test; Z = −2.06; *p* = 0.04) and a lower frequency of intercourse (Z = −5.17; *p* < 0.0001). Similarly, among these respondents, sexual life satisfaction was lower both over their lifetime (Z = −4.51; *p* < 0.0001) and over the last 12 months (Z = −5.34; *p* < 0.0001). In the group with clinically significant dysfunctions, a significantly higher prevalence of each symptom of sexual dysfunction was observed. Interestingly, no such difference was found among the observed symptoms of partner dysfunction, apart from premature ejaculation.

A comparative analysis was conducted across groups based on the prevalence of individual sexual dysfunction symptoms compared to scores on individual FSFI domains and the total FSFI score. Similar correlations were found to those described in the previous paragraph, as presented in Table 4. Regarding partner dysfunctions, premature ejaculation in the partner observed by the respondent was significantly associated with lower scores in the FSFI satisfaction domain (Z = 3.5; *p* = 0.0004) and the FSFI orgasm domain (Z = 2.6; *p* = 0.008). While initial univariate analysis suggested potential associations with the total FSFI score (Z = 2.1; *p* = 0.04) and the FSFI pain domain (Z = 2.2; *p* = 0.03), these relationships did not retain statistical significance after applying the Benjamini–Hochberg correction for multiple comparisons. This indicates that the negative association between partner’s premature ejaculation, as observed by the respondent, is specific to the domains of satisfaction and orgasm, rather than affecting the woman’s global sexual function. Apart from premature ejaculation observed by the respondents, other symptoms of partner sexual dysfunction did not show a statistically significant impact on the respondents’ quality of sexual life.

The number of co-occurring symptoms of dysfunction (as detailed in Table 3) strongly negatively correlated (Spearman’s rank correlation; *p* < 0.0001) with the total FSFI score (r = −0.49) and with each FSFI domain: desire (r = −0.31), arousal (r = −0.35), lubrication (r = −0.28), orgasm (r = −0.42), satisfaction (r = −0.32), and pain (r = −0.38). The number of different sexual dysfunction symptoms in the partner, as observed by the respondents, correlated quite strongly with the FSFI satisfaction domain (r = −0.3; *p* < 0.0001) and weakly with the FSFI orgasm domain (r = −0.14; *p* < 0.05). In cases where the partner experienced premature ejaculation observed by the respondent, a higher number of co-occurring sexual dysfunction symptoms were observed in the respondent (Mann–Whitney U Test; z = 4.4; *p* < 0.0001), as well as a simultaneously higher number of other co-occurring sexual dysfunction symptoms in the partner (z = 7.4; *p* < 0.0001). No relationship was observed between age, place of origin, or place of residence and the FSFI score.

### 3.8. Statistical Analysis of the Factors Affecting the FSFI Score

Finally, to address the third research question, we performed multivariable regression to identify independent predictors of the FSFI total score.

#### 3.8.1. Univariate Analysis

The results of univariate analysis presented in Table 5 showed that FSFI scores differed significantly based on the frequency of sexual intercourse, sexual satisfaction (in the last 12 months and lifetime), number of sexual partners, relationship status, presence of sexual dysfunction symptoms in the respondent, number of symptoms, and specific sexual difficulties (orgasm, lubrication, pain, decreased interest). Additionally, the presence of premature ejaculation in the partner (observed by the respondent) was associated with FSFI scores (*p* < 0.05).

#### 3.8.2. Multivariable Analysis Setup

To identify independent predictors of the FSFI score (dependent variable), variables with *p* < 0.15 in the univariate analysis were initially considered for the model. These included: frequency of sexual intercourse, sexual satisfaction (last 12 months and lifetime), number of sexual partners, relationship status, presence/number of dysfunction symptoms, specific sexual difficulties, and observed partner’s premature ejaculation. Prior to the final analysis, collinearity diagnostics were performed. To ensure model stability and avoid multicollinearity, “lifetime sexual satisfaction” was excluded in favor of “satisfaction in the last 12 months.” Table 6 presents the independent variable values.

#### 3.8.3. Multivariable Regression Result

Three statistically significant independent factors were identified in the final regression equation, as shown in Table 7. According to the standardized partial regression coefficients, the strongest independent predictors associated with the total FSFI score were as follows:

(1) Frequency of sexual intercourse (positive association);
(2) Sexual satisfaction in the last 12 months (positive association);
(3) Number of symptoms of sexual dysfunction (negative association).

**Table 7:** Multivariable regression results of factors affecting the FSFI score; SE—standard errors for coefficients.

Independent Variable	Coefficient	SE	t	*p* Value
Constant term	18.93	1.40	13.52	<0.00001
Frequency of sexual intercourse	2.02	0.31	6.53	<0.00001
Sexual satisfaction (last 12 months)	1.25	0.34	3.64	0.0003
Number of symptoms of sexual dysfunction	−1.04	0.23	−4.45	<0.00001

## 4. Discussion

The relationship between being in a stable partnership and women’s sexual quality of life demonstrates complex patterns. In our study, women in stable relationships reported greater sexual satisfaction. Higher scores were observed, particularly in the orgasm and arousal domains, as well as in overall FSFI scores, though without a significant reduction in the likelihood of sexual dysfunctions. Similarly, a study among German medical students found that having a stable partner was significantly associated with higher FSFI total scores [20]. A study conducted in Turkey showed that the quality of sexual life is significantly correlated with sexual compatibility with a partner [21].

The results of our study confirm the well-documented association between sexual activity frequency, subjective sexual satisfaction, and outcomes in individual FSFI domains, particularly regarding overall sexual life satisfaction. An international study encompassing diverse populations (Europe, North and South America) demonstrated that women engaging in sexual activity ≥ 11 times per month reported significantly higher levels of desire, arousal, orgasm, and satisfaction compared to those with lower sexual frequency [22]. In the study population, women with FSFI scores in the risk range for FSD (i.e., at or below the established cutoff) reported less frequent sexual activity, especially in cases of diminished sexual interest and difficulties achieving orgasm. A study conducted in South Korea found that lower coital frequency constituted a significant, independent risk factor for FSD [23].

The application of the five-point Likert scale in our study made it possible to capture women’s subjective satisfaction with their sexual lives in a simple and clinically intuitive way, complementing the information obtained through the FSFI questionnaire. In our sample, this single-item rating showed strong associations with FSFI total and domain scores, suggesting that it may function as a pragmatic, preliminary screening indicator of women’s sexual well-being in similar populations. However, this measure has not been formally validated and should not be viewed as a diagnostic tool or a substitute for a comprehensive assessment of sexual functioning. Its potential utility lies in helping clinicians to quickly identify women who may benefit from a more detailed sexual health evaluation, rather than in providing definitive conclusions about sexual dysfunction. In line with previous research, our findings indicate that sexual activity frequency may play an important role in sexual quality of life among women in our sample. When considered together with psychological and relational factors, frequency of intercourse appears to be one of several behavioral markers associated with better sexual functioning, with possible implications for sexual health promotion and early identification of difficulties. Nevertheless, given the non-representative, self-selected nature of the sample and the cross-sectional design, these patterns should be interpreted cautiously and confirmed in larger, representative studies.

Numerous studies have consistently shown that sexual dysfunctions are prevalent among women of reproductive age, frequently manifesting as reduced FSFI scores across the domains of desire, arousal, lubrication, orgasm, and satisfaction. In our cohort, we also observed a high prevalence of self-reported sexual difficulties (75% of participants reporting at least one symptom), particularly concerning orgasmic difficulties, dyspareunia, and diminished sexual interest. However, it is crucial to distinguish between isolated symptoms and clinically significant dysfunction; when applying the FSFI cutoff score, 35.2% of respondents obtained scores at or below the threshold, indicating an increased risk of FSD rather than a confirmed diagnosis. This aligns with a systematic review and meta-analysis including 215,740 women, chronic sexual dysfunction affects approximately 41% of women of reproductive age, with sexual desire disorder being the most reported (28%) [24]. Similarly, a study conducted in the northeastern Black Sea region of Turkey reported that as many as 70.9% of married women aged 18–50 years scored below the FSFI cutoff, particularly in the domains of desire and arousal [25]. In a cross-sectional diagnostic study in Thailand including 346 women, the overall prevalence of FSD was 40.2%, with the most frequently reported issues being lack of sexual desire, orgasmic disorders, and difficulties with arousal and lubrication [26].

In our study, we observed a correlation between the presence of dysfunction-level FSFI scores and a lower number of sexual partners within the preceding 12 months, a finding not reflected in other scientific reports. A large cohort study from Slovenia, including 605 women, did not demonstrate a direct association between partner number and FSFI domain scores [27]. Likewise, a study among Polish women with type 1 diabetes did not reveal a significant relationship between lifetime number of partners and FSFI outcomes [28].

The results of this study suggest that, in our sample, women who perceived premature ejaculation (PE) in their male partners tended to report lower overall sexual functioning as measured by the FSFI, as well as lower scores in specific domains of sexual life. Reduced total FSFI scores among these participants are consistent with the possibility that partner-related difficulties may be linked to broader challenges in women’s sexual well-being. However, after rigorous statistical correction for multiple comparisons (FDR), the presence of perceived partner PE was significantly associated specifically with lower scores in the FSFI satisfaction and orgasm domains, but not with the total FSFI score or the pain domain. This suggests that while PE (observed by the partner) may not necessarily be accompanied by global sexual dysfunction or pain in the woman, it is strongly linked to unmet sexual needs and difficulties in reaching the culmination of the sexual response cycle due to shortened intravaginal latency time. These observations are partially consistent with previous reports, although our findings are conservative due to the statistical correction applied. In a study by Canat et al. (2018), female partners of men with PE had significantly lower total FSFI scores [29]. Similarly, a meta-analysis of a Chinese cohort reported lower scores across all domains [30]. Our study, by applying strict control for Type I errors, highlights that the strongest link exists for satisfaction and orgasm. Importantly, the presence of observed PE was associated with a higher number of concomitant sexual dysfunction symptoms in the respondent. This supports the view that PE may serve as a marker of broader challenges in the couple’s sexual life, consistent with Hobbs et al., who reported that female partners of men with PE experienced multiple dysfunctions significantly more often than controls [31].

Of particular interest is that a study utilizing behavioral therapy (“stop–start technique”) in men with PE demonstrated significant improvements in their partners’ sexual function, with increases in overall FSFI scores and marked improvements across all domains, including desire, arousal, satisfaction, and reduced pain [32]. Taken together, these findings suggest that PE may have important implications for both partners. This emphasizes the need for an integrated management approach combining medical treatment with psychosexual interventions to improve relationships and quality of life.

Our analysis further showed that among male sexual dysfunctions reported by respondents, premature ejaculation showed the clearest association with specific female sexual difficulties. With respect to sexual satisfaction, the number of observed male sexual dysfunctions exhibited the strongest negative association, consistent with findings from Maseroli et al. [33]. A weaker yet notable correlation with the orgasm domain suggests that orgasmic difficulties in women may represent a downstream correlate of partner-related dysfunction. Nonetheless, the non-representative, self-selected nature of our sample and the reliance on women’s perceptions of their partners’ difficulties mean that these conclusions must be interpreted with caution.

## 5. Limitations

Despite the important findings of this study, several limitations should be considered. First, the study used an online, convenience, self-selected sample recruited via Facebook student groups. The participants, therefore, do not constitute a representative sample of Polish female university students or of the general female population. Facebook is relatively less popular among younger generations, and the mean age in our sample (26.2 years) suggests an overrepresentation of older or non-traditional students. As a result, younger undergraduate women may be underrepresented, and the findings should not be generalized to all female students.

Second, the cross-sectional design precludes causal inference. Although we observed associations between perceived partner sexual dysfunctions and women’s sexual functioning, the direction of these relationships cannot be established; pre-existing relational or sexual difficulties may contribute both to reduced sexual satisfaction and to the perception of partner dysfunctions. Sexual functioning was assessed with the FSFI, which covers only the preceding four weeks, so dysfunction-level scores may reflect transient rather than chronic problems. In this study, the FSFI cutoff was used as a screening threshold indicating an increased risk of female sexual dysfunction, not as a definitive clinical diagnosis, and isolated sexual difficulties reported outside the FSFI were treated as self-reported symptoms rather than diagnostic entities.

Third, all data were obtained through self-report. Information on male partners’ sexual dysfunctions, including premature ejaculation, was based solely on women’s responses and thus reflects their subjective perceptions rather than clinical diagnoses or male self-report using validated instruments, introducing potential perceptual and relational bias. Overall sexual satisfaction was measured with a non-validated single-item 5-point scale, which may provide a clinically relevant preliminary screening signal but cannot be considered a diagnostic tool or a substitute for comprehensive standardized assessment and requires further validation. The sociodemographic questionnaire was author-designed and has not been formally validated, which may affect measurement precision.

Finally, this study should be understood as exploratory. The patterns we observed are therefore best viewed as hypothesis-generating and should be replicated and refined in future studies with larger, more diverse, and methodologically rigorous samples.

## 6. Conclusions

1. In this self-selected, non-representative sample of female university students, over 75% reported at least one self-perceived sexual difficulty, while 35.2% obtained FSFI scores at or below the cutoff, indicating an increased risk of FSD rather than a confirmed diagnosis. Sexual difficulties in this relatively young group were particularly common in the domains of desire and orgasm, suggesting a need for early educational and preventive interventions. Women’s sexual functioning and satisfaction were associated not only with individual factors but also with relational and partner-related ones.
2. Women who perceived premature ejaculation in their male partners tended to report lower sexual satisfaction and poorer orgasmic functioning, and a higher number of perceived partner dysfunctions correlated with greater deficits in women’s sexual functioning. Although causality cannot be inferred and these data reflect women’s perceptions, the findings support a relational, couple-centered perspective in clinical assessment.
3. The single-item five-point scale of overall sexual satisfaction showed strong correlations with FSFI scores, indicating potential usefulness as a simple preliminary screening question for subjective sexual satisfaction. However, as this measure has not been formally validated, it cannot be regarded as a diagnostic tool or a substitute for standardized instruments and requires further psychometric evaluation.

## Figures and Tables

**Table 1:** Characteristics of sociodemographic factors of the study group. (LS) = Likert scale: 1–5.

		%
N		199
Mean age ± SD		26.2 ± 6.2
Mean year of studying		3.2 ± 1.8
Branch of study:	Artistic	5.5%
	Economics and management	8.5%
	Natural sciences/Agriculture	1.0%
	Medical	36.7%
	Sciences/Polytechnic/IT/Mining and metallurgy	15.1%
	Sports	2.5%
	Humanities/Pedagogy/Law/Religion	18.6%
	Other	12.1%
Place of origin:	village	21.6%
	<20 k inhabitants	14.1%
	20–50 k inhabitants	13.1%
	50–100 k inhabitants	14.6%
	100–200 k inhabitants	18.6%
	>200 k inhabitants	18.1%
Place of residence:	village	9.5%
	<20 k inhabitants	5.0%
	20–50 k inhabitants	7.5%
	50–100 k inhabitants	9.5%
	100–200 k inhabitants	26.6%
	>200 k inhabitants	41.7%
Where do you currently live?	In an apartment with friends	15.1%
	In a dorm	8.0%
	Alone	13.6%
	With parents	29.6%
	In an apartment with a partner	33.7%
Do you have children?	No	87.4%
	Yes	12.6%
Do you live by the principles of your religion:	No	77.9%
	Yes	22.1%
How much do you live by the principles of your religion? (LS)		3.4 ± 0.9

**Table 2:** Characteristics of the population in terms of gender identification, sexual orientation, and being in a relationship.

		%
Sexual orientation	Asexual	1.0%
	Bisexual	16.6%
	Heterosexual	78.4%
	Homosexual	2.0%
	I do not know	1.0%
	Pansexual	1.0%
The gender you identify with is different from your birth certificate	Yes	4.5%
	No	95.5%
Do you have a regular sexual partner?	Yes (one)	81.9%
	Yes (many)	1.5%
	No	16.6%
Are you in a regular relationship:	Yes, Married	10.1%
	Yes, Partner	45.2%
	No	44.7%
How often do you have sex?	<1/month	16.8%
	1–2/month	22.5%
	1–2/week	37.7%
	3–4/week	14.1%
	>4/week	8.9%

**Table 3:** Characteristics of the study population according to the presence or absence of clinically significant sexual dysfunctions according to the FSFI. (LS) = Likert scale: 1–5; (NS)—non-significant.

				FSFI		
			All	Current Dysfunction	No Dysfunction	*p*
			269	70	199	
			100%	35.2%	62.8%	
Number of sexual partners (lifetime)			3.8 ± 5.5	3.2 ± 4.3	4.2 ± 6.1	NS
Number of sexual partners (last 12 months)			1.2 ± 0.8	1.0 ± 0.4	1.3 ± 0.9	0.04
Sexual satisfaction (lifetime) (LS)			3.8 ± 1.0	3.4 ± 1.1	4.1 ± 0.8	<0.0001
Sexual satisfaction (last 12 months) (LS)			3.8 ± 1.2	3.2 ± 1.2	4.1 ± 1.0	<0.0001
Frequency of sexual intercourse (LS)			2.6 ± 1.3	2.0 ± 1.2	3.0 ± 1.2	<0.0001
Have you noticed any of the following abnormalities in your sexual life in the last 12 months:	Decreased interest in sexuality	Yes	41.4%	66.7%	27.9%	<0.0001
		No	58.6%	33.3%	72.1%	
	Problem with lubrication of the genital tract during intercourse	Yes	31.0%	45.6%	23.3%	0.001
		No	69.0%	54.4%	76.7%	
	Pain during intercourse	Yes	45.5%	60.9%	37.8%	0.002
		No	54.5%	39.1%	62.2%	
	Problem with achieving orgasm during intercourse	Yes	53.1%	66.2%	46.5%	0.01
		No	46.9%	33.8%	53.5%	
	Problem with achieving orgasm during masturbation	Yes	24.1%	35.3%	18.1%	0.008
		No	75.9%	64.7%	81.9%	
In the last 12 months, have you noticed the following irregularities in your PARTNER’S sexual life:	Decreased interest in sexuality	Yes	22.3%	24.2%	21.4%	NS
		No	77.7%	75.8%	78.6%	
	Erection problem	Yes	11.3%	11.3%	11.3%	NS
		No	88.7%	88.7%	88.7%	
	Pain during intercourse	Yes	6.0%	5.0%	6.6%	NS
		No	94.0%	95.0%	93.4%	
	Premature ejaculation	Yes	23.9%	32.3%	19.8%	0.05
		No	76.1%	67.7%	80.2%	
	Problem with achieving orgasm during intercourse	Yes	17.8%	13.1%	20.2%	NS
		No	82.2%	86.9%	79.8%	

**Table 4:** The presence or lack of statistical significance in the case of comparison using the Mann–Whitney U Test—comparison of the results of the FSFI scale and individual FSFI domains between groups with or without a given declared sexual dysfunction in the respondent or her partner; (NS)—non-significant; (NS*)—did not retain statistical significance after applying the Benjamini–Hochberg correction for multiple comparisons.

	FSFI Score	FSFI Domain					
		D1	D2	D3	D4	D5	D6
		Desire	Arousal	Lubrication	Orgasm	Satisfaction	Pain
Self-reported dysfunction symptom (YES/NO)							
Decreased interest in sexuality	*<0.0001*	*<0.0001*	*<0.0001*	*0.0005*	*<0.0001*	*<0.0001*	*0.0002*
Problem with lubrication of the genital tract during intercourse	*<0.0001*	*0.003*	*0.0001*	*<0.0001*	NS	*0.01*	*<0.0001*
Pain during intercourse	*<0.0001*	*0.005*	*0.003*	*0.03*	*<0.0001*	*0.01*	*<0.0001*
Problem with achieving orgasm during intercourse	*<0.0001*	NS	*0.02*	NS	*<0.0001*	*0.0005*	NS
Problem with achieving orgasm during masturbation	*0.0009*	NS	*0.01*	NS	*<0.0001*	*0.005*	NS
Observed Dysfunction Symptom in Partner: (YES/NO)							
Decreased interest in sexuality	NS	NS	NS	*0.02*	NS	*0.01*	NS
Problem with achieving orgasm during intercourse	NS	NS	NS	NS	NS	NS	NS
Erection problems	NS	*NS*	NS	NS	NS	*0.011*	NS
Premature ejaculation	NS*	NS	NS	NS	*0.008*	*0.0004*	*NS*
Pain during intercourse	NS	NS	NS	NS	NS	0.04	NS

**Table 5:** Univariate analysis of the factors affecting the FSFI score.

Characteristics	r	t	*p* Value
Sexual satisfaction (last 12 months)	0.54	9.02	<0.0001
Frequency of sexual intercourse	0.51	8.42	<0.0001
Sexual satisfaction (lifetime)	0.45	7.09	<0.0001
Being in a stable relationship	0.24	3.48	0.0006
Number of sexual partners (last 12 months)	0.2	2.91	0.004
Number of sexual partners (lifetime)	0.14	2.05	0.04
Presence of premature ejaculation in the partner	−0.15	−2.13	0.03
Problem with achieving orgasm during masturbation	−0.24	−3.42	0.0008
Problem with achieving orgasm during intercourse	−0.29	−4.23	<0.0001
Problem with lubrication of the genital tract during intercourse	−0.35	−5.14	<0.0001
Presence of symptoms of any dysfunction	−0.37	−5.63	<0.0001
Pain during intercourse	−0.37	−5.4	<0.0001
Decreased interest in sexuality	−0.45	−7	<0.0001
Number of symptoms of sexual dysfunction	−0.49	−7.85	<0.0001

**Table 6:** Assignment sheet of the influencing factors of the FSFI score; * observed by respondent in the last 12 months.

9	Assignment
Sexual satisfaction (last 12 months)	Initial data (Likert score: 1–5)
Sexual satisfaction (lifetime)	Initial data (Likert score: 1–5)
Frequency of sexual intercourse	1 = <1/month 2 = 1–2/month 3 = 1–2/week 4 = 3–4/week 5 = >4/week
Being in a stable relationship	0 = No 1 = Yes
Number of sexual partners (last 12 months)	Initial data
Number of sexual partners (lifetime)	Initial data
Presence of premature ejaculation in the partner *	0 = No 1 = Yes
Problem with achieving orgasm during masturbation *	0 = No 1 = Yes
Problem with achieving orgasm during intercourse *	0 = No 1 = Yes
Problem with lubrication of the genital tract during intercourse *	0 = No 1 = Yes
Presence of symptoms of any dysfunction *	0 = No 1 = Yes
Pain during intercourse *	0 = No 1 = Yes
Decreased interest in sexuality *	0 = No 1 = Yes
Number of symptoms of sexual dysfunction *	Initial data

## Data Availability

The data supporting the findings of this study are not publicly available due to the anonymous nature of the web-based survey and ethical restrictions, but may be provided by the corresponding author upon reasonable request.

## Abbreviations

The following abbreviations are used in this manuscript:

FSFI	Female Sexual Function Index
WHO	World Health Organization
DSM	Diagnostic and Statistical Manual of Mental Disorders
FSD	Female Sexual Dysfunction
